# Supplementary material for: Enhanced homeostatic sleep response and decreased neurodegenerative proteins in cereblon knock-out mice
Source: Commun Biol. 2024 Sep 30;7:1218. doi: 10.1038/s42003-024-06879-y (PMC11442454; doi:10.1038/s42003-024-06879-y)
Supplement: Supplementary file 2 — Supplementary Information [file 42003_2024_6879_MOESM2_ESM.pdf]

Enhanced Homeostatic Sleep Response and Decreased Neurodegenerative Proteins in Cereblon Knock-Out Mice

Jun-Hyung Jung[1†], Jinhong Kim[2†], Uroos Akber[1], Na Young Lee[1], Jeong-won Baek[1], Jieun Jung[2], Mincheol Park[2], Jiseung Kang[2], Seungje Jeon[1,3], Chul-Seung Park[1*], Tae Kim[2*]

[1]Laboratory of Molecular Neurobiology, School of Life Sciences, Gwangju Institute Science and Technology (GIST), Gwangju, 61005, Republic of Korea
[2]Laboratory of Translational Neuroscience, Department of Biomedical Science and Engineering, Gwangju Institute Science and Technology (GIST), Gwangju, 61005, Republic of Korea
[3]Department of Ophthalmology, University of Texas Southwestern Medical Center, Dallas, Texas, United States

[†]These authors contributed equally.
[*]Correspondence should be addressed to T.K. (tae-kim@gist.ac.kr) and C.S.P.(cspark@gist.ac.kr)

SUPPLEMENTARY INFORMATION: Supplementary Figures 1-4, Supplementary Table 1

Supplementary Figures

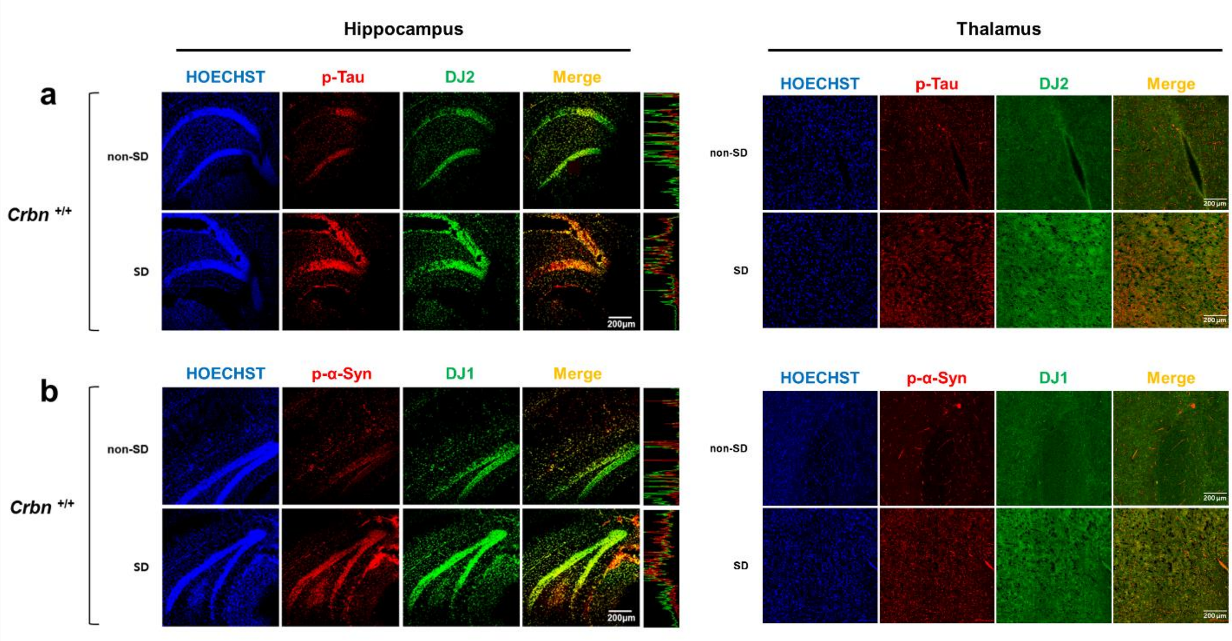

Supplementary Figure 1. Effects of sleep deprivation (SD) on phosphorylation states of neuropathological proteins and expression of molecular chaperones. Immunohistochemistry analysis of the hippocampal region and thalamus in *Crbn*$^{+/+}$ mice after 6-h of SD was performed using confocal microscopy. Tissues were stained with **a** Hoechst (blue), phosphorylated Tau (p-Tau, red), and DJ2 (green) or **b** Hoechst (blue), phosphorylated α-Synuclein (p-α-Syn, red), and DJ1 (green) antibodies.

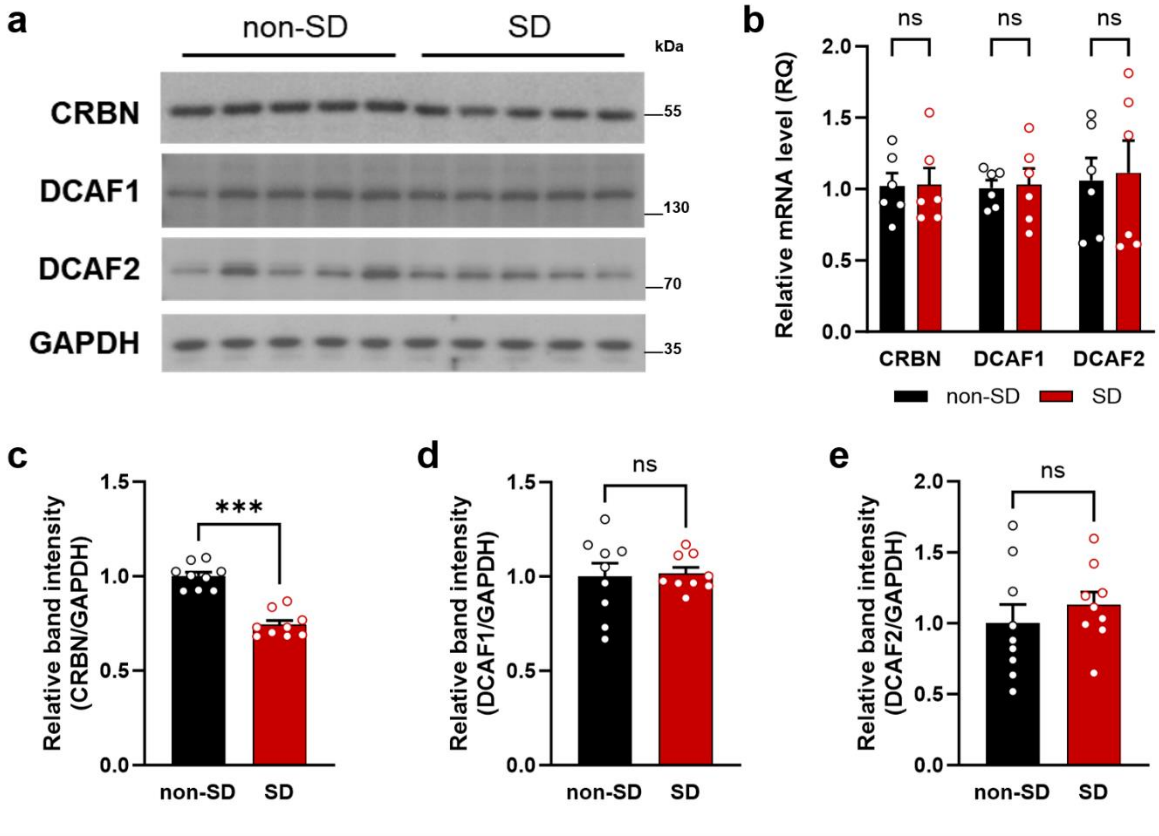

Supplementary Figure 2. Protein and mRNA levels of substrate receptors of CUL4 E3 ligase in *Crbn*$^{+/+}$ mice after sleep deprivation (SD). Brains of *Crbn*$^{+/+}$ mice were collected after 6-h of SD and compared with those of non-SD *Crbn*$^{+/+}$ mice (n = 9). **a** Protein levels of CUL4 E3 ligase substrate receptors CRBN, DCAF1, and DCAF2 were measured by western blot analysis. **b** Total RNA from *Crbn*$^{+/+}$ mice was isolated after SD, and quantitative real-time PCR analysis was performed to measure mRNA of *Crbn*, *Dcaf1*, and *Dcaf2*. Band intensity of **c-e** CRBN, DCAF1, and DCAF2 relative to GAPDH was determined by densitometry. Data are presented as mean ± SEM. **P* < 0.05, ***P* < 0.01, ****P* < 0.001.

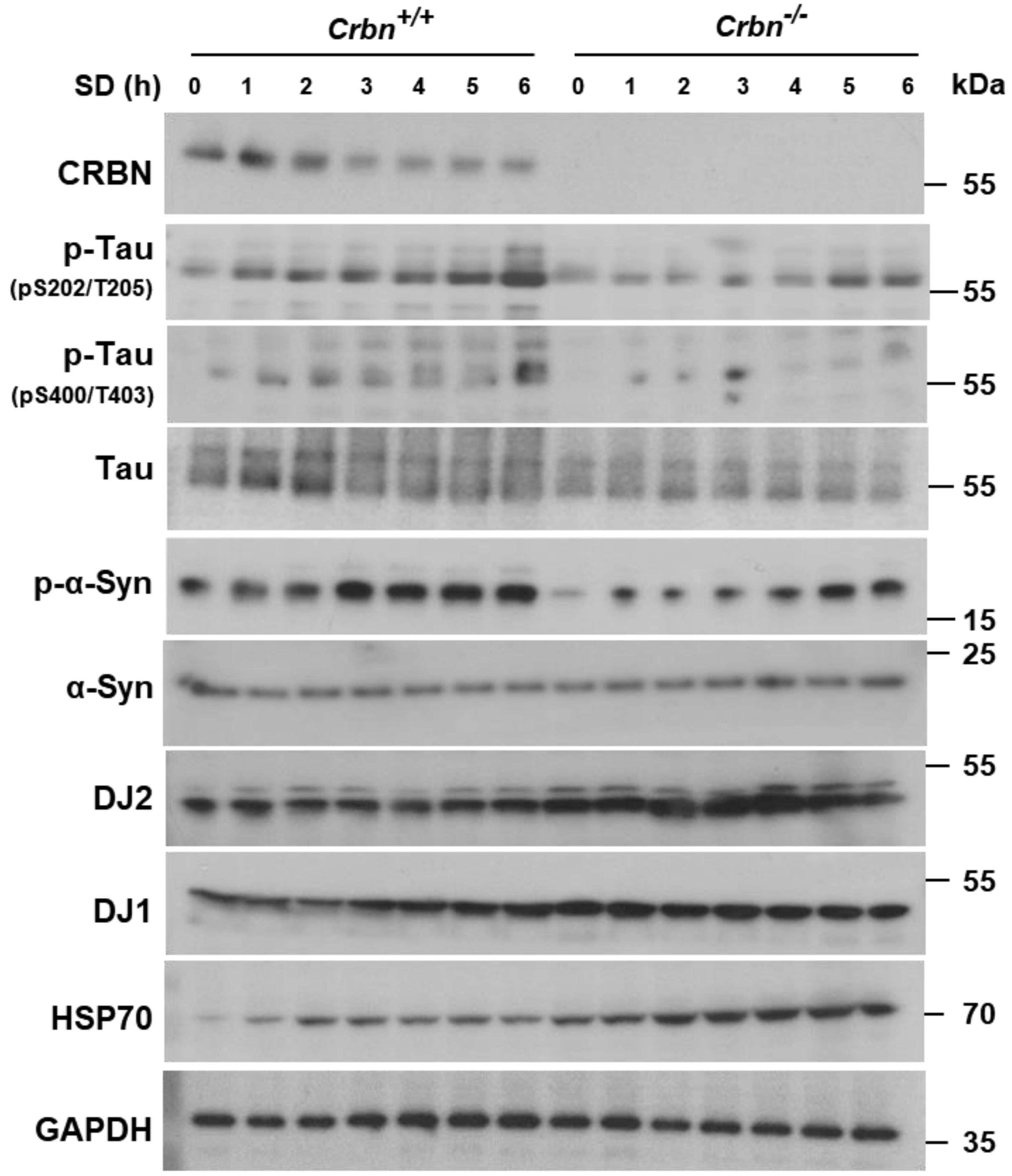

Supplementary Figure 3. Altered protein expression in *Crbn*$^{-/-}$ mice during 6 h timely sleep deprivation (SD). Brains of *Crbn*$^{-/-}$ mice were collected at different time points during SD and compared with those from non-SD *Crbn*$^{-/-}$ mice (n = 3). Levels of each protein were detected by specific antibodies. GAPDH was used as a loading control.

Supplementary Figure 4. Uncropped blots of figures

Figure 1a

Long exposure (showing protein ladder)

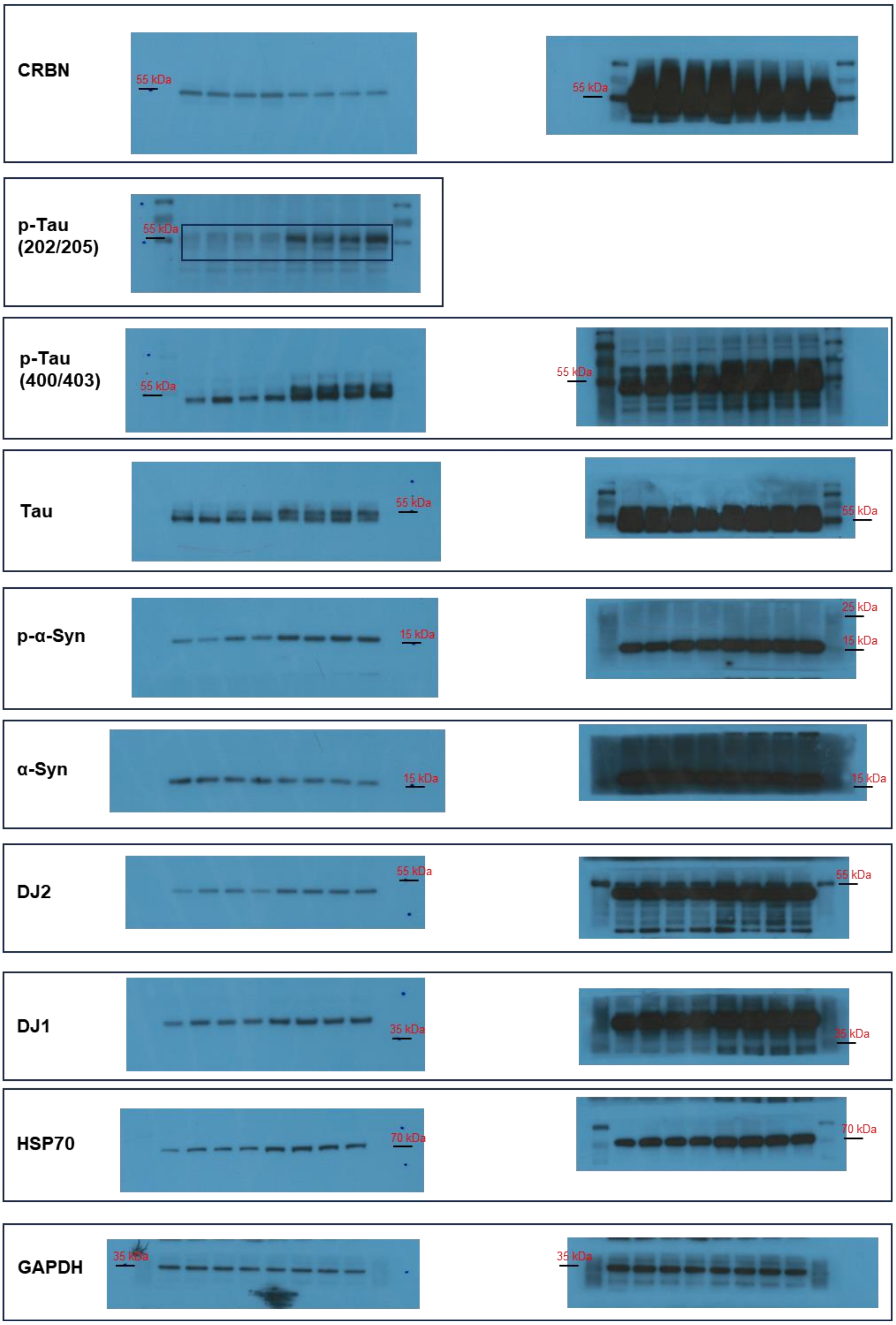

Supplementary Figure 4. Uncropped blots of figures (Continued)

Figure 2a

Long exposure (showing protein ladder)

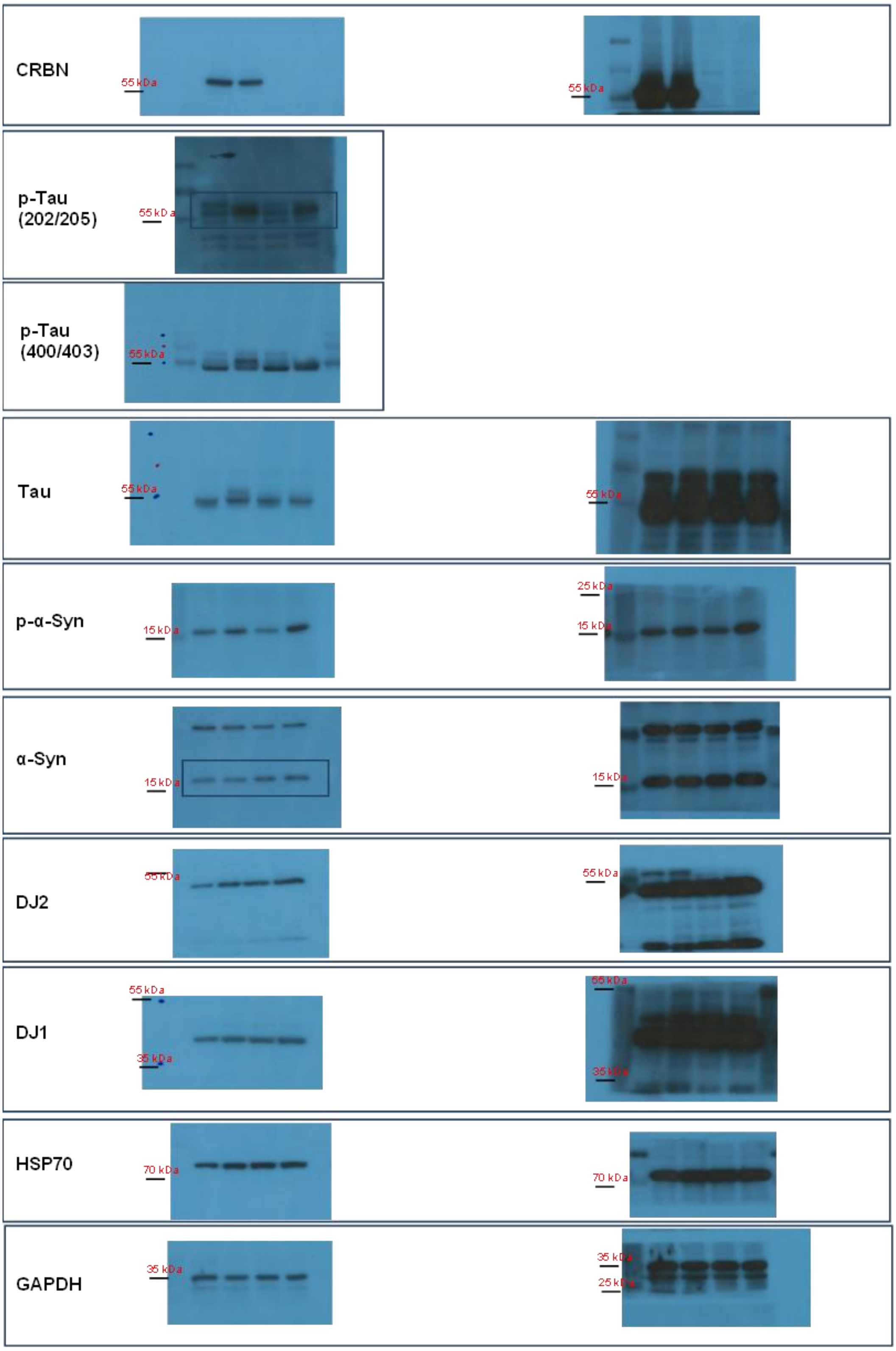

Supplementary Figure 4. Uncropped blots of figures (Continued)

Figure 4a

Long exposure (showing protein ladder)

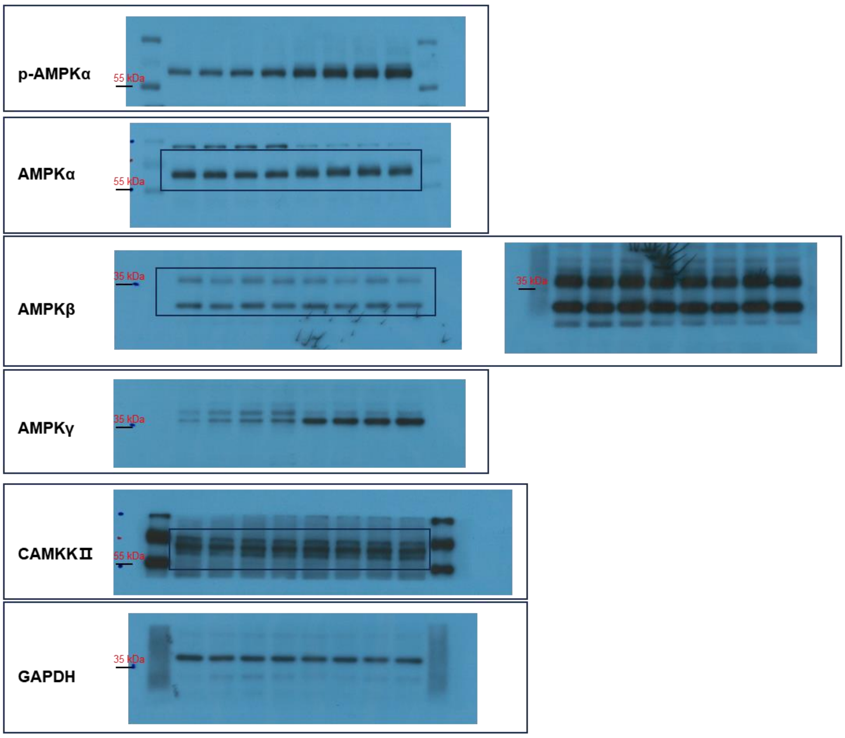

Figure 4c

Long exposure (showing protein ladder)

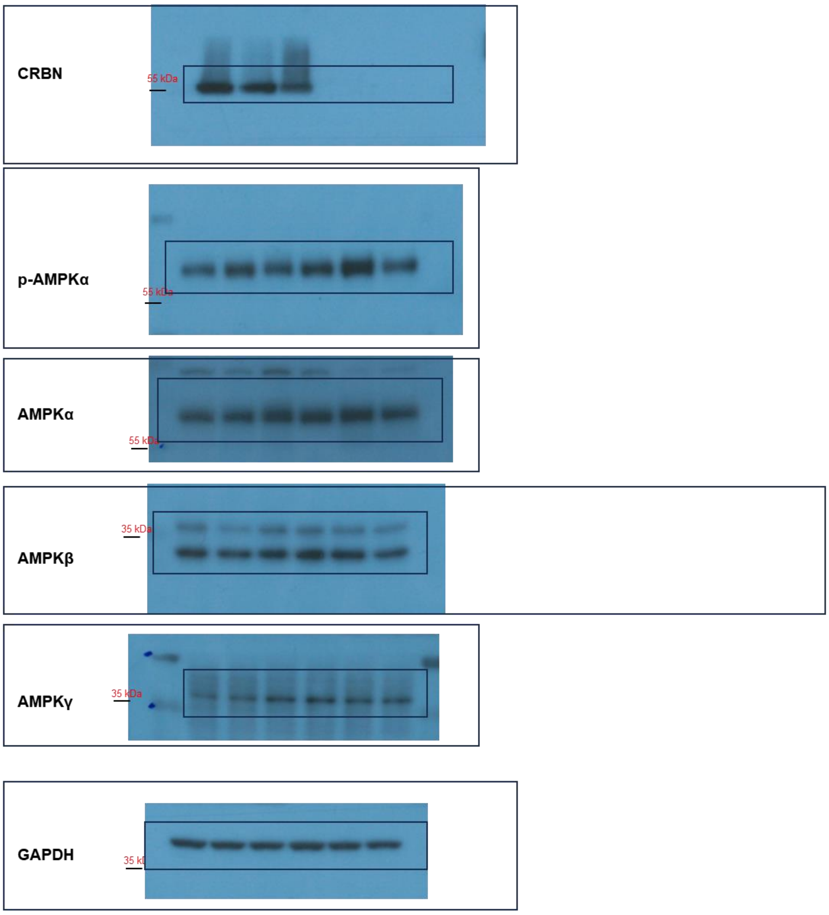

Supplementary Figure 4. Uncropped blots of figures (Continued)

Figure 4e

Long exposure (showing protein ladder)

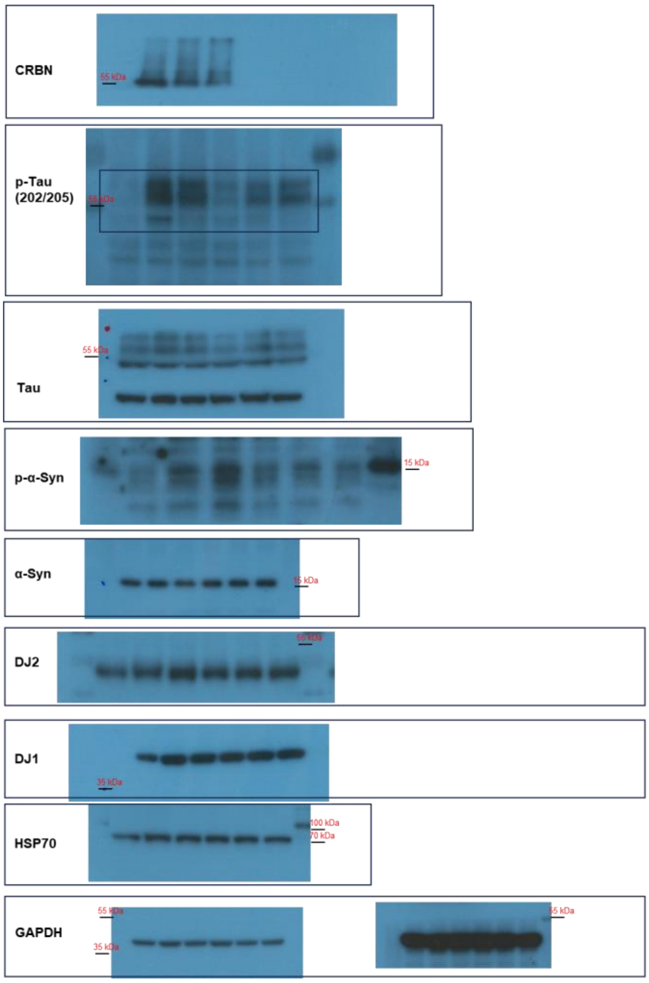

Supplementary Figure 2a

Long exposure (showing protein ladder)

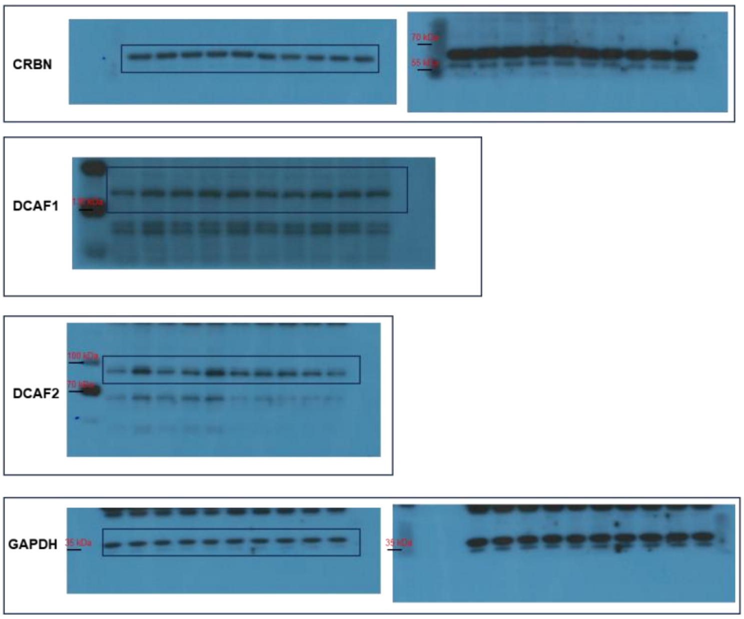

Supplementary Figure 4. Uncropped blots of figures (Continued)

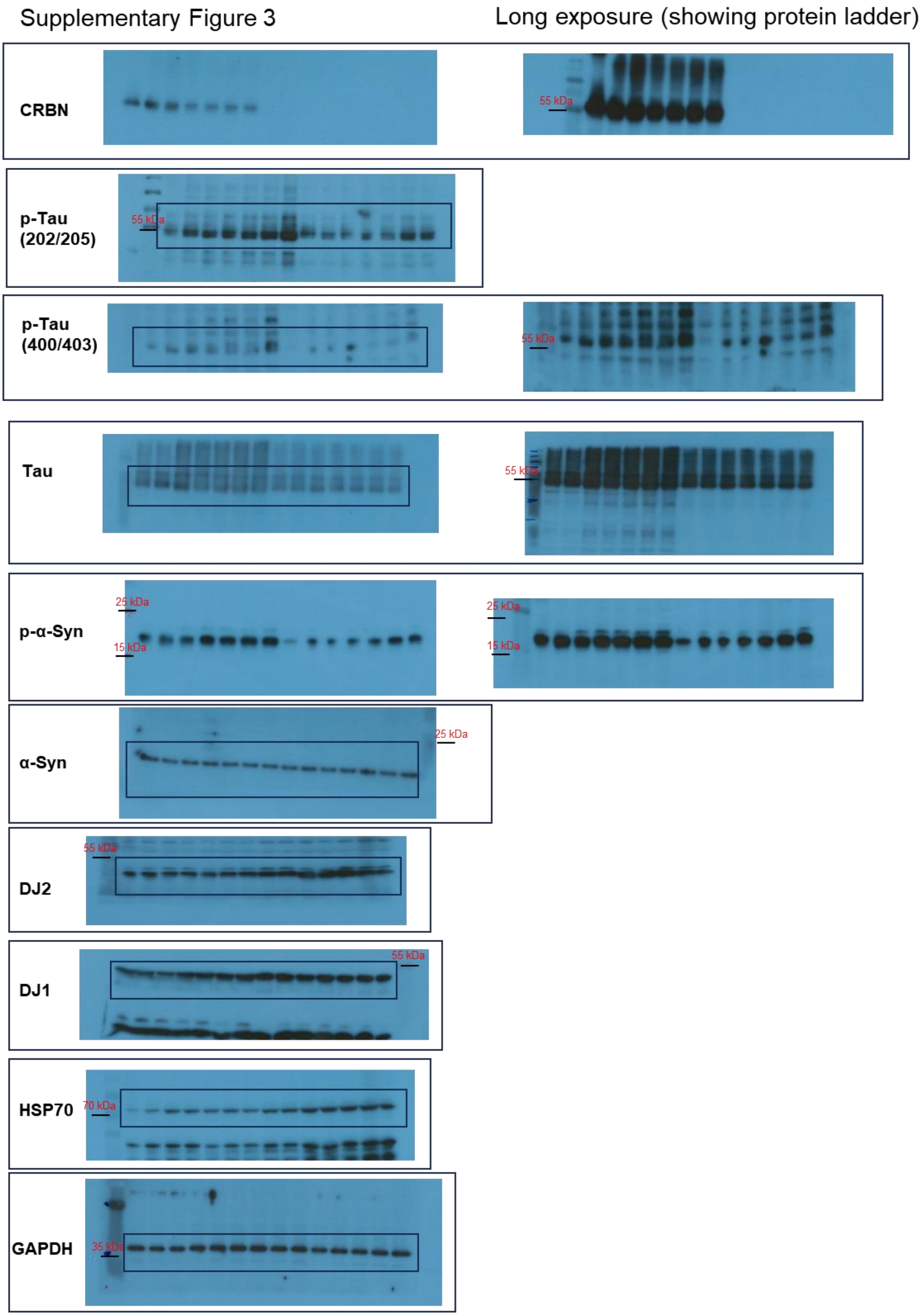

Supplementary Table 1. Antibodies and Reagents used in this study

Antibodies / Reagents	Company	Catalog number
CRBN	CST	71810
CRBN	Abnova	H00051185-B01P
p-Tau (S202/T205)	Thermo Fisher Scientific	MN1020
p-Tau (S400/T403)	CST	11837 (Discontinued)
Tau	Abcam	ab254150
p-α-Synuclein	Abcam	ab184674
α-Synuclein	Abcam	ab1903
DJ2	Abcam	ab126774
DJ1	Abcam	ab231577
HSP70	CST	4872
GAPDH	Abfrontier	LF-PA0018
p-AMPKα	CST	2535
AMPKα	Invitrogen	AOH1332
AMPKβ	CST	4150
AMPKγ	Abcam	ab32508
CAMKKII	Invitrogen	PA5-78909
DCAF1	Invitrogen	PA5-99967
DCAF2	Abcam	ab72264
HRP-conjugated Goat Anti-Rabbit IgG Secondary Antibody	Jackson ImmunoResearch Laboratories	111-035-003
Anti-Mouse IgG (H+L), HRP Conjugate Secondary Antibody	Jackson ImmunoResearch Laboratories	115-035-003
Alexa Fluor-488 anti-rabbit	Thermo Fisher Scientific	A11034
Alexa Fluor-594 anti-mouse	Thermo Fisher Scientific	A11005
HOECHST	Abcam	ab145597
Compound C	Sigma-Aldrich	171260
