# Supplementary material for: Enhanced homeostatic sleep response and decreased neurodegenerative proteins in cereblon knock-out mice
Source: Commun Biol. 2024 Sep 30;7:1218. doi: 10.1038/s42003-024-06879-y (PMC11442454; doi:10.1038/s42003-024-06879-y)
Supplement: Supplementary file 3 — Description of Additional Supplementary File [file 42003_2024_6879_MOESM3_ESM.pdf]

Description Of Additional Supplementary File

File name: Supplementary Data 1

Description: The source data of the graphs in the paper
